# Supratentorial hydrocephalus with an anti‐glial fibrillary acidic protein (GFAP) antibody‐positive status: A case report

**DOI:** 10.1111/cns.13969

**Published:** 2022-09-08

**Authors:** Lei Tang, Le Zhang, Min Zhang, Yi Zeng, Ye Li

**Affiliations:** ^1^ Department of Neurology The Fourth Hospital of Changsha Changsha China; ^2^ National Clinical Research Center for Geriatric Disorders, Xiangya Hospital Central South University Changsha China; ^3^ Department of Neurology Multi‐Modal Monitoring Technology for Severe Cerebrovascular Disease of Human Engineering Research Center Changsha China; ^4^ Department of Neurology, Second Xiangya Hospital Central South University Changsha China

**Keywords:** anti‐GFAP‐antibody, biomarker, case report, supratentorial hydrocephalus

Dear Editor,

Currently, there are only a few reports on patients with an anti‐glial fibrillary acidic protein (GFAP) antibody‐positive status, and the underlying pathophysiological mechanism and clinical significance remain controversial, warranting further clarification. Caution should be exercised when determining the clinical significance of an antibody. In this study, we report a rare case of supratentorial hydrocephalus with an anti‐GFAP‐antibody positive status and review relevant literature with the hope to improve clinicians' understanding of GFAP antibodies.

A 58‐year‐old man was admitted to the hospital owing to dizziness and headache for 1 month, which was aggravated with hiccups, unstable walking, and slow response for half a month. The patient had a history of trauma before onset (a stick hit to the top of his head but no coma at that time). There were no obvious abnormalities in the findings of general internal medicine physical examination. Physical examination by a neurological specialist revealed a slightly declining memory of recent and distant events. The results of the bilateral finger nose, calcaneal tibia, rotation, and Romberg tests were positive.

Lumbar puncture was performed, which showed a pressure of 180 mmHg and a protein level of 1.08 g/L in cerebrospinal fluid (CSF). Cytology findings showed a normal white blood cell count, which was dominated by lymphocytes, with individual neutrophils and eosinophils, and plasma cells, suggesting an inflammatory response, and red blood cell (RBC) phagocytes were seen in the CSF. In CSF neuron antigen spectrum antibody test (−). With the consent of the patient and his family members, the serum and CSF samples were collected from the patient for the detection of four central nervous system (CNS) demyelinating antibodies and autoimmune encephalitis antibodies. Results of CSF and serum testing for CNS‐demyelinating antibodies revealed a positive anti‐GFAP‐IgG antibody status in the patient's serum (titer of 1:100) and CSF (titer of 1:3.2) (Figure 1). Multiple tests for potential neoplastic causes yielded negative results. Contrast enhancement on brain magnetic resonance imaging (MRI) suggested supratentorial hydrocephalus (Figure 2), with no other obvious abnormalities. Cervicomedullary MRI showed no obvious abnormalities.

**FIGURE 1:**
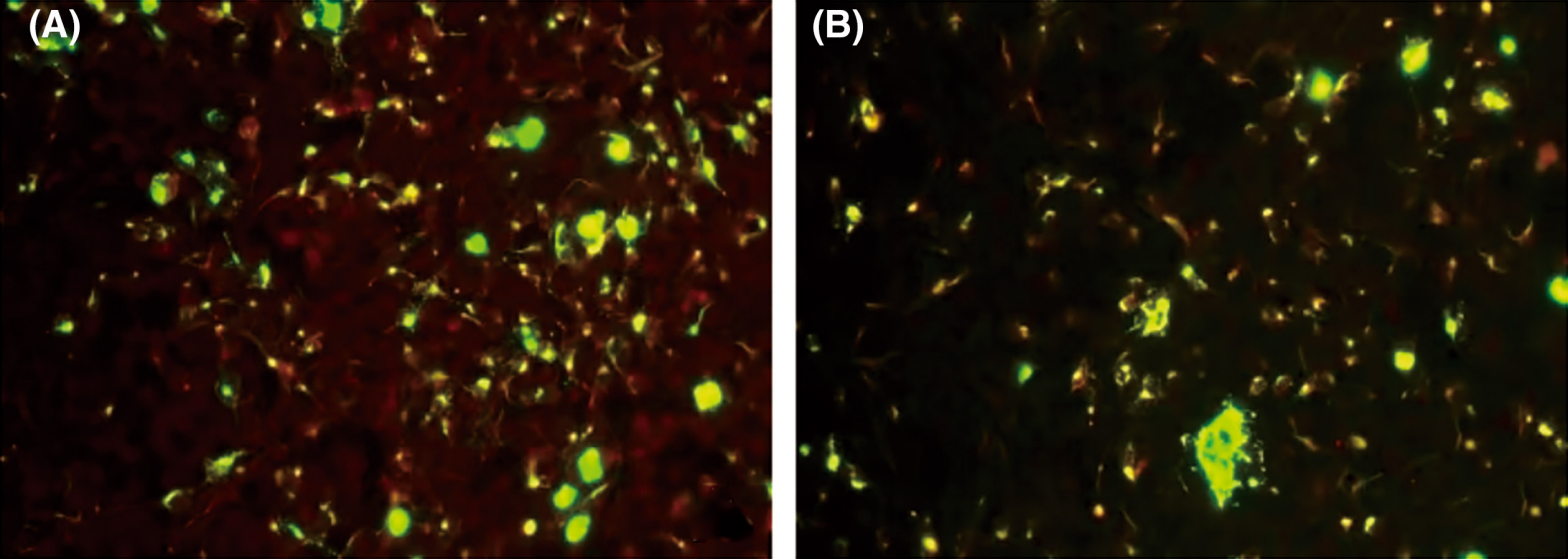
Results of anti‐GFAP antibody detection in the serum and CSF samples of the patient by cell‐based assay. Panel A: anti‐GFAP antibody was positive (titer 1: 100) in the serum and Panel B: anti‐GFAP antibody was positive (titer was 1:3.2) in the CSF. CSF, cerebrospinal fluid; GFAP, glial fibrillary acidic protein.

**FIGURE 2:**
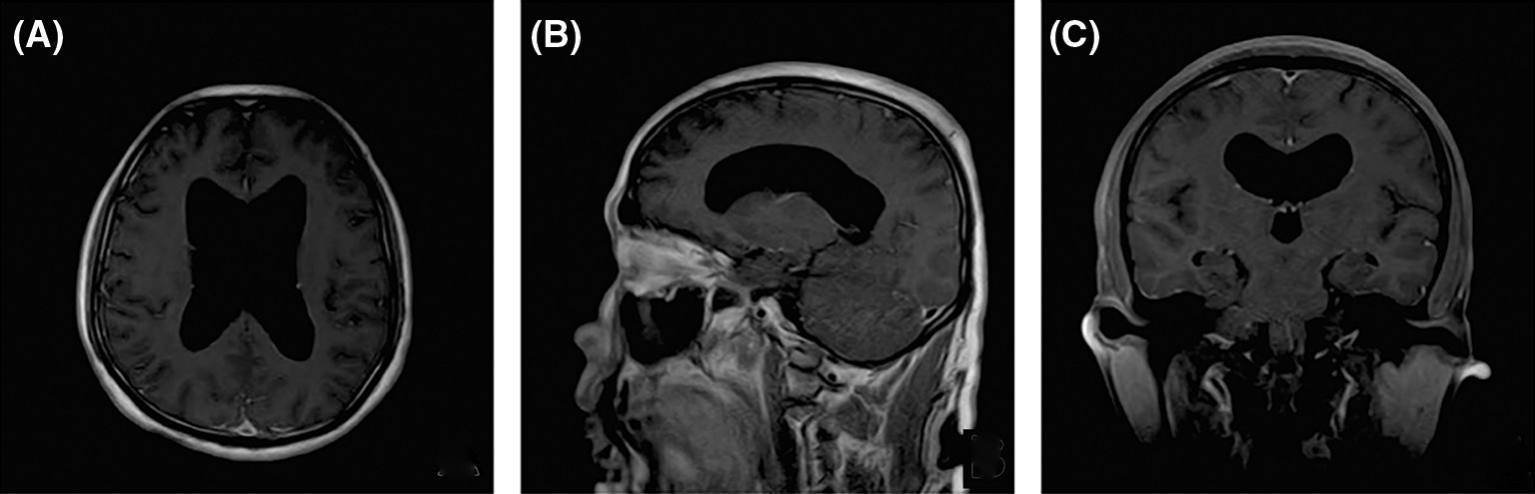
Findings of brain magnetic resonance imaging. Panel A: axial strengthening; Panel B: sagittal enhancement; and Panel C: coronal enhancement. The panels show supratentorial hydrocephalus.

Subsequently, a lumbar puncture test was performed again and approximately 30 ml of CSF was released. The patient's hiccups disappeared and his walking instability gradually improved the next day. At that point, his cognitive function, walking instability, dizziness, and headache had improved. A ventriculoperitoneal shunt was placed considering a positive drainage test result. The patient's clinical symptoms significantly improved after the operation and no hormone shock therapy was administered. At the 1‐month follow‐up, the patient showed no obvious clinical symptoms.

GFAP is an intermediate filament protein between the microfilaments and large microtubules of astrocytes in the CNS. It is a pathological target and biomarker of astrocytes and plays an important role in the pathogenesis of CNS injury and related diseases.^1^, ^2^, ^3^ The inflammatory reaction that can occur in patients with a positive anti‐GFAP‐IgG antibody status mainly involves the meninges, brain parenchyma, spinal cord, spinal meninges, and optic nerve.^2^, ^4^ The corresponding clinical phenotypes are diverse and there are certain difficulties in diagnosis and treatment.

GFAP astrocytic disease (GFAP‐A) is an inflammatory autoimmune disease of the CNS as proposed by Fang et al.^1^ At present, GFAP immunoglobulin G (GFAP‐IgG) is considered a specific biomarker for the diagnosis of the disease, and a positive GFAP‐IgG status in the CSF has high specificity and sensitivity. The disease is clinically rare, with more than 300 cases reported worldwide.^5^, ^6^ Currently, there is no unified diagnostic standard for GFAP‐A; hence, many doubts are troubling clinicians, such as how to classify and diagnose patients with atypical clinical manifestations and positive GFAP antibodies in the CSF.

Anti‐GFAP‐IgG antibody‐related diseases may not be independent disease diagnoses, and the clinical significance of antibodies needs to be evaluated carefully. At present, it is not clear whether the anti‐GFAP‐IgG antibody is a marker or pathogenic antibody. It has been reported that the anti‐GFAP‐IgG antibody is not a pathogenic antibody but is a potential marker of astrocyte destruction caused by cytotoxic T cell‐mediated autoimmune responses.^7^

In this case, the patient had influenza‐like prodromal symptoms after the stick hit the top of the head; therefore, it is speculated that infection may be one of the causes of GFAP‐IgG antibody production. Infections activate the immune system and during immune‐mediated astrocyte destruction, the body releases chemokines, recruits inflammatory cells, and causes the spread of immune attacks to the normal nervous system.^8^ There may also be some undiscovered plasma membrane protein‐directed IgG, which may trigger autoimmune events and destroy the function of astrocytes, and GFAP autoimmunity occurs as a secondary event.^9^ It is also worth noting that there was a small amount of subarachnoid hemorrhage, which occurred because the stick hit the top of the head (no hemorrhage was found on craniocerebral computed tomography but RBC phagocytosis could be seen on CSF cytology). Considering the possibility of traumatic brain injury, it can cause complex pathological reactions in the brain, including secondary injury, which is mainly driven by astrocytes, microglia, and immune cells infiltrating from the peripheral circulation, resulting in persistent neuronal and vascular dysfunction.^10^ Reactive astrocytes are characterized by the structural and functional transformation of the resting astrocytes, including the increased expression of intermediate filaments such as GFAP.^10^ Some studies have shown that inflammation is an important factor in the occurrence of hydrocephalus after subarachnoid hemorrhage in which the formation of astrocytes and glial scars plays an important role in the inflammatory response of the CNS,^3^, ^10^ considering that anti‐GFAP‐IgG antibody may be a biomarker of immune‐inflammatory processes.

In summary, the pathophysiological mechanism of the anti‐GFAP‐IgG antibody is unclear, and a unified diagnosis and treatment standard has not been established, which needs to be further clarified and solved. This case presents hydrocephalus with a positive GFAP antibody status after mild traumatic subarachnoid hemorrhage, which has rarely been reported. This article discusses the possible mechanism of its formation combined with the literature and enriches the spectrum and clinical phenotype of GFAP‐IgG antibody‐related diseases, with the hope of providing some experience and help to clinicians.

## AUTHOR CONTRIBUTIONS

L.T. wrote the manuscript. Y.L., M.Z., Y.Z., and L.Z. analyzed the clinical data and provided professional comments to the manuscript. All authors have read and approved the final manuscript.

## CONFLICT OF INTEREST

The authors declare that the research was conducted in the absence of any commercial or financial relationships that could be construed as a potential conflict of interest.
